# Duty Factor Is a Viable Measure to Classify Spontaneous Running Forms

**DOI:** 10.3390/sports7110233

**Published:** 2019-11-10

**Authors:** Aurélien Patoz, Cyrille Gindre, Adrien Thouvenot, Laurent Mourot, Kim Hébert-Losier, Thibault Lussiana

**Affiliations:** 1 Research and Development Department, Volodalen Swiss SportLab, 1860 Aigle, Switzerland; cyrille@volodalen.com; 2 Research and Development Department, Volodalen, 39134 Chavéria, France; adrien@volodalen.com (A.T.); thibault@volodalen.com (T.L.); 3 Research Unit EA3920 Prognostic Markers and Regulatory Factors of Cardiovascular Diseases and Exercise Performance, Health, Innovation platform, University of Fanche-Comté, 25000 Besançon, France; laurent.mourot@univ-fcomte.fr; 4 Division for Physical Education, Tomsk Polytechnic University, 634050 Tomsk, Russia; 5 Adams Centre for High Performance, Division of Health, Engineering, Computing and Science, Te Huataki Waiora School of Health, University of Waikato, Tauranga 3116, New Zealand; kim.hebert-losier@waikato.ac.nz

**Keywords:** running, biomechanics, running form

## Abstract

Runners were classified using two different methods based on their spontaneous running form: (1) subjectively using the V^®^score from the Volodalen^®^ scale, leading to terrestrial and aerial groups; and (2) objectively using the duty factor (DF), leading to high (DF_high_) and low (DF_low_) DF groups. This study aimed to compare these two classification schemes. Eighty-nine runners were divided in two groups using the V^®^score (VOL groups) and were also ranked according to their DF. They ran on a treadmill at 12 km·h^−1^ with simultaneous recording of running kinematics, using a three-dimensional motion capture system. DF was computed from data as the ratio of ground contact time to stride time. The agreement (95% confidence interval) between VOL and DF groups was 79.8% (69.9%, 87.6%), with relatively high sensitivity (81.6% (68.0%, 91.2%)) and specificity (77.5% (61.6%, 89.2%)). Our results suggest that the DF and V^®^score reflect similar constructs and lead to similar subgroupings of spontaneous running form (aerial runners if DF < 27.6% and terrestrial runners if DF > 28.8% at 12 km·h^−1^). These results suggest that DF could be a useful objective measure to monitor real-time changes in spontaneous running form using wearable technology. As a forward-looking statement, spontaneous changes in running form during racing or training could assist in identifying fatigue or changes in environmental conditions, allowing for a better understanding of runners.

## 1. Introduction

Walking is a gait pattern often modelled as an “inverted pendulum” in which the height of the center of mass (COM) is maximal at mid-stance. On the other hand, running is a “bouncing” gait modelled as a mass on a spring with the height of the COM being minimal at mid-stance. The spring–mass model considers the relationship between vertical displacement and bouncing frequency [1]. This model is based on the assumption that the supporting leg behaves like a spring during stance and that each stance is separated by a period of no contact with the ground or flight. Novacheck postulated that the presence of this flight phase (*t_f_*) allowed to distinguish walking from running gaits [2]. However, it has been shown that quadrupeds, such as elephants, run without a *t_f_* [3]. Similarly, several bipeds use an intermediate gait, termed grounded running by Andrada et al. [4], that shares the characteristics of the spring–mass model of running during stance, but is deprived of a *t_f_* [4,5].

Recently, a study [5] observed modifications of biomechanical and metabolic parameters in runners that performed instructed grounded running at a slow speed (2.1 m·s^−1^). When using this intermediate form of gait instead of their natural running gait, runners decreased their musculoskeletal load but increased their energy expenditure [5]. In this particular style of gait, the presence of a *t_f_* does not appropriately discriminate walking and running. That said, relatively slow locomotive speeds seem necessary to achieve a run without a *t_f_*. Due to the decrease of the musculoskeletal load, this intermediate gait form could be useful in long distance and ultra-running events to off-load the musculoskeletal system. The existence of such a range of slow-speed spontaneous running gait forms imply variable *t_f_* between runners. Even at higher speeds, Ogueta-Alday et al. [6] reported large standard deviation in *t_f_* values at various speeds, highlighting the variability amongst runners in this particular metric. Therefore, it becomes possible to distinguish between different running patterns and classify runners along a continuum at any given running speed on the basis of *t_f_* or *t_f_* reliant metrics.

Depending on speed, we have observed differences of 10% to 30% in *t_f_* of two different spontaneous running forms [7,8,9]. In these aforementioned studies, we have used a global subjective rating scale (named Volodalen^®^ scale) to classify runners into two main categories, based on scores along a continuum (V^®^score). Aerial runners (AER) favor vertical oscillations and longer *t_f_* as opposed to terrestrial runners (TER) who prefer propelling their body forward and use shorter *t_f_* [9]. These groups are termed VOL groups in what follows. In addition, a recent study [10] has observed similar between group differences in *t_f_* when runners were classified using their duty factor (DF) values compared to the VOL classification. The DF, defined as the ratio of the ground contact time (*t_c_*) to the stride time (the sum of *t_c_* and swing time (*t_s_*)), allows to rank runners using an objective temporal measure of the running form and can be used to classify runners in separate DF groups. The runners with a high DF (DF_high_) in this particular study [10] reduced work against gravity to promote forward progression (longer *t_c_*) and limit their *t_f_,* whereas those with a low DF (DF_low_) had a more symmetrical running step and favored a shorter *t_c_* and longer *t_f._* Moreover, we noted in these studies [7,8,9,10] 5% to 15% differences in *t_c_* depending on the running speed within their respective VOL and DF groups, again highlighting the potential ability of *t_c_* or *t_c_* reliant metrics to distinguish between the spontaneous running forms. 

Both VOL and DF classification methods tend to highlight similar differences in temporal parameters, such as *t_c_* and *t_f_* within their respective groups. However, the existence of a relationship between the groups created using the Volodalen^®^ scale and the DF metric has not yet been considered. Therefore, the purpose of this study was to compare these two different classification methods in analyzing running gait. As distinct *t_c_* and *t_f_* are observed between TER and AER runners, we anticipated an agreement in the classification of runners between VOL and DF groups. Moreover, this study sought to define DF ranges at a particular running speed for which an agreement between VOL and DF groups should be expected.

## 2. Materials and Methods

### 2.1. Subjects

Sixty-five males (age: 38.3 ± 9.4 y, height: 176.9 ± 6.8 cm, body mass: 72.0 ± 9.7 kg, and running distance: 44.5 ± 25.4 km·week^−1^) and 24 females (age: 36.2 ± 8.1 y, height: 165.6 ± 6.4 cm, body mass: 59.3 ± 6.6 kg, and running distance: 33.2 ± 18.9 km·week^−1^) voluntarily participated in this study. All participants had been running regularly for at least two years at the time of their study participation. Inclusion criteria were good self-reported general health and no current or recent (<3 months) lower-extremity injury. The university’s institutional review board approved the study protocol prior to participant recruitment (CPP: 2014-A00336-41), which was conducted in accordance with international ethical standards [11], and adhered to the Declaration of Helsinki of the World Medical Association.

### 2.2. Experimental Procedure

Each participant completed one experimental session. Testing was performed under similar environmental conditions (23 ± 2 °C and 45 ± 7% relative humidity). All participants were advised to avoid strenuous exercise the day before the test. After providing written informed consent, retro-reflective markers were positioned on participants to assess their running biomechanics (details on marker position below). For each participant, a 5-s standing static trial using a standard reference position was recorded on a treadmill (Medic 2850, Technologies Machines Spéciales, Champs-sur-Yonne, France) for calibration purposes. Then, a 10-min run was performed on the treadmill. Participants were permitted to select and modify their running speed from 8 to 12 km·h^−1^ during the 10 min, and were requested to maintain the 12 km·h^−1^ for at least the last minute to allow group comparisons at a common speed. Three-dimensional (3D) kinematic data were collected during the last 30 s of the running trial. All participants were familiar with running on a treadmill as part of their usual training program and wore their habitual running shoes during testing.

### 2.3. Subjective Assessment of Running Gait

During the last minute of the 10-min run, a running coach with more than 5 years of experience using the Volodalen^®^ scale focused on the overall movement pattern of participants. The coach focused on five key elements—(A) vertical oscillation of the head, (B) antero-posterior motion of the elbows, (C) vertical pelvis position at ground contact, (D) antero-posterior foot position at ground contact, and (E) strike pattern [7,8]. Each element was scored from one to five, leading to a global subjective score (V^®^score) that represents the spontaneous running form of participants. This V^®^score ultimately allows the classification of runners into two different categories: TER (V^®^score ≤ 15) or AER (V^®^score > 15), which has been shown to be a reliable method to assess running form [12]. Both intra- (expert) and inter-rater (expert versus novice) absolute reliabilities of V^®^score values were reported to be adequate, with the coefficient of variations being 6.1 ± 7.0% and 6.6 ± 6.5%, respectively, with no large systematic bias between V^®^scores (paired *t*-test: *P* = 0.864 and 0.248, respectively) [12].

### 2.4. Data Collection

Whole-body 3D kinematic data were collected at the maximal sampling frequency of our operating system (179 Hz) using eight infrared Oqus 500+ cameras and the Qualisys Track Manager software version 2018.1, build 4100 (Qualisys AB, Göteborg, Sweden). Forty-five and forty-one retro-reflective markers of 12 mm in diameter were used for static and running trials, respectively. They were affixed to the skin and shoes of individuals over anatomical landmarks using double-sided tape, following standard guidelines from the Project Automation Framework Running package [13].

The 3D marker data were exported in .c3d format and processed in Visual3D Professional software version 6.01.12 (C-Motion Inc., Germantown, Maryland, USA). More explicitly, the 3D marker data were low-pass filtered at 20 Hz using a fourth-order Butterworth filter. From the marker set, a full-body biomechanical model with 15 rigid segments was constructed, with each segment tracked using six degrees of freedom. Segments included the head, upper arms, lower arms, hands, thorax, pelvis, thighs, shanks, and feet. In Visual3D, segments were treated as geometric objects. Segments were assigned inertial properties and COM locations based on their shape [14] and were attributed a relative mass based on standard regression equations [15]. Whole-body COM location was calculated from the parameters of all 15 segments.

Running events were derived from the kinematic data. More explicitly, mid-toe and mid-foot landmarks were created midway between the first and fifth toe markers, and the heel marker and mid-toe landmark, respectively. The mid-toe landmark was rescaled by subtracting its respective global minimum. Moreover, heel and mid-toe accelerations were calculated as the second derivative of the heel marker and mid-toe landmark, respectively. As for each running trial, footstrike was defined as the first acceleration spike between the heel marker and mid-toe landmark acceleration spikes. Toe-off was defined as the instance when the mid-toe reached 1 cm on ascent. All events were verified to ensure correct identification and were manually adjusted when required.

*t_s_* and *t_c_* were defined as the time from toe-off to footstrike and from footstrike to toe-off of the same foot, respectively. Values for *t_s_* and *t_c_* were calculated based on footstrike and toe-off events, and DF was calculated as follows [16]

$$\mathrm{DF}=\;\frac{t_{c}}{t_{c}+t_{s}}$$

### 2.5. Objective Assessment of Running Gait

The 89 participants were ranked according to their DF computed from the data collected during the last 30 s of their 10-min run at 12 km·h^−1^. To avoid a potential bias due to sex, the number of males and females was matched between the VOL and DF groups. To do so, the number of males and females was counted in TER (*N*_TER,M_ and *N*_TER,F_) and AER (*N*_AER,M_ and *N*_AER,F_). Then, the DF values of all participants were arranged in decreasing order and by sex. DF_high_ was composed of the *N*_TER,M_ and *N*_TER,F_ with the largest DF values within males and females, respectively. The remaining constituted DF_low_, i.e., the *N*_AER,M_ and *N*_AER,F_ with the smallest DF values within males and females, respectively. Data from all 89 participants were included in the statistical analysis.

### 2.6. Statistical Analysis

Descriptive statistics are presented using mean ± standard deviation (S.D.). Normality and homogeneity of data were verified using Shapiro–Wilk and Levene tests, respectively. To compare participant characteristics between groups, non-parametric Wilcoxon–Mann–Whitney tests were used when normality was violated, Welch-tests for normally distributed but inhomogeneous data, and bilateral Student’s *t*-tests were used otherwise. Moreover, sensitivity and specificity of the agreement between DF and VOL groups, defined as the proportion of actual DF_high_ runners that have been attributed as TER runners and of non-DF_high_ runners that have been attributed as non-TER runners, respectively, were calculated. The 95% confidence intervals (lower, upper) of the agreement between VOL (TER, AER) and DF (DF_high_, DF_low_) groups, as well as the sensitivity and specificity values, were estimated using binomial exact calculation. Statistical analysis was done using customized scripts in R 3.5.0 (The R Foundation for Statistical Computing, Vienna, Austria) with a level of significance set at α ≤ 0.05.

## 3. Results

### 3.1. VOL and DF Characteristics

The subjective assessment using the V^®^score led to 49 TER and 40 AER runners, whereas the objective assessment using DF led to 49 DF_high_ and 40 DF_low_ runners (Table 1). Most characteristics were similar within VOL and DF groups, although AER and DF_low_ runners ran more than TER and DF_high_ runners. The V^®^score was significantly lower in DF_high_ runners, and DF was significantly greater in TER runners.

### 3.2. VOL and DF Agreement

Seventy-one TER and AER runners were attributed to their expected DF group, resulting in a 79.8% (69.9%, 87.6%) agreement. Therefore, 18 participants were “misclassified” and belonged to the alternative group (Table 2). Sensitivity and specificity were 81.6% (68.0%, 91.2%) and 77.5% (61.6%, 89.2%), respectively.

There were no significant differences in participant characteristics between runners in their expected versus the unexpected DF group for both TER and AER runners (Table 3).

### 3.3. VOL and DF Agreement Ranges

**Figure 1:**
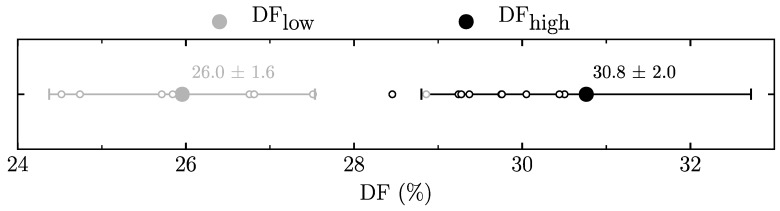
Duty factors (DF) for high (DF_high_, black) and low (DF_low_, gray) duty factor groups. Black-outlined circles denote the aerials runners assigned to DF_high_ expected to belong to DF_low_ (n = 9). Gray-outlined circles denote the terrestrial runners assigned to DF_low_ expected to belong to DF_high_ (n = 9).

Based on our results, we were able to define DF ranges at 12 km·h^−1^ for which the DF_high_ and DF_low_ groups corresponded to the TER and AER groups (Figure 1). The upper bound for DF_low_ was 27.6% (defined as mean + S.D., i.e., 26.0 + 1.6%), while the lower bound for DF_high_ was 28.8% (defined as mean—S.D., i.e., 30.8 – 2.0%). As such, there was a DF interval between 27.6% to 28.8% where AER and TER groups were not defined as clearly. The DF of most of the “misclassified” participants (14 out of 18) was situated within the intervals designated as the DF_low_ and DF_high_ groups (Figure 1). Moreover, the average DF of TER and AER runners “misclassified” as DF_low_ (26.4%) and DF_high_ (29.7%) were contained within the defined DF_low_ and DF_high_ intervals, respectively. 

## 4. Discussion

In accordance with our hypothesis, the agreement between the VOL and DF groups was 79.8%. We defined that a runner was attributed to the AER group if the DF value was lower than 27.6% and to the TER group if the DF value was higher than 28.8% at 12 km·h^−1^.

The DF values in our population were in line with those previously reported in the literature at similar running speeds [16,17]. Based on the DF, which is an objective measure of the running form, a runner was attributed to its expected DF group (i.e., DF_high_ for a TER runner and DF_low_ for an AER runner) in 79.8% of cases, leading to a specificity of 81.6% and sensitivity of 77.5%. This level of agreement between the VOL and DF groups stemmed from the fact that AER runners favor vertical bounce, leading to a longer *t_f_* and a shorter *t_c_* compared to TER runners who favor horizontal propulsion, longer *t_c_*, and shorter *t_f_* [7,9]. Thereby, even if a runner is not classified to a VOL group, based on the *t_c_* and *t_f_* values, but using the V^®^score (which is independent of *t_c_* and *t_f_*), group attribution is strongly linked to these temporal parameters and the DF. Therefore, based on our definitions of DF ranges for which the DF_high_ and DF_low_ groups are expected to be similar to the TER and AER groups, respectively, the DF measure can provide a relatively accurate classification of runners to the VOL groups in the absence of a Volodalen rater. 

However, one must remember that the running form of individuals varies along a continuum. Therefore, even if our definition of DF ranges for the DF_high_ and DF_low_ groups would allow the classification of a runner in a particular VOL group at 12 km·h^−1^, there exists a DF range from 27.6% to 28.8% where the two groups are quite similar along this continuum. This 1.2% gap between the upper bound of DF_low_ and the lower bound of DF_high_ highlights the existence of mixed runners. Similarly, the V^®^score threshold value of 15 allows the classification of runners in TER and AER categories, but again, bearing in mind that VOL groups are not necessarily dichotomous and that the V^®^score rather positions runners along a continuum. Appreciating these characteristics of spontaneous running forms of individuals might prove to be important in individualizing their training programs.

A 100% agreement was not obtained between the VOL and DF classifications. Nevertheless, we observed that the TER and AER runners belonging to their alternative DF group are located, in most of the cases (14 out of 18), within our defined DF_low_ and DF_high_ groups, based on their DF values. In addition, no significant differences for TER and AER runners attributed to DF_high_ and DF_low_ groups were noted (Table 3). These observations reflect the individualized nature of spontaneous running forms and movement patterns, where a subset of individuals did not respond to the defined paradigms. Of note, however, is the slightly greater V^®^score of “misclassified” TER runners (DF_low_: 12.0 vs. DF_high_: 11.3) and their greater training (DF_low_: 5.0 h and 46 km per week vs. DF_high_: 3.9 h and 35 km per week), although not reaching statistical significance. We can speculate that these more trained runners follow the current trend of favoring forefoot striking, leading to a lower DF than expected. Indeed, forefoot strike pattern is associated with a shorter *t_c_* and a longer *t_f_* [6], thus, influencing DF values without an obvious impact on subjective evaluations based on the V^®^score. Similarly, when considering AER runners, a slightly higher V^®^score was obtained for expected DF_low_ than non-expected DF_high_ runners (V^®^score: 19.1 vs. 18.6), although not reaching statistical significance. Moreover, expected DF_low_ runners trained more (running time: 5.2 vs. 3.9 h·week^−1^ and running distance: 50 vs. 33 km·week^−1^) and were younger (age: 37 vs. 41 y), despite not reaching statistical significance. Therefore, we can speculate that these runners had a lower DF than expected due to the fact that younger runners have a longer *t_f_* and similar *t_c_* than older runners [18]. However, this argument has to be taken with caution, given the lack of significant difference in terms of age between DF_low_ and DF_high_ runners (*P* = 0.384). Further research is needed to better understand the underlying reason for these discrepancies between the VOL and DF groups, and could include the use of multivariate analyses. 

The objective quantification of the running form using the DF has become much easier to perform. Indeed, with the rapid evolution of technology, DF can be easily measured with a simple to use, inexpensive, and accurate device such as an inertial measurement unit. Such units have been proven to be able to detect stance duration during running gait [19]. Moreover, such units are not restricted to indoor laboratory environments, but allow outdoor recordings, and could be used to measure the DF of outdoors runners and classify them in their corresponding equivalent VOL groups. However, DF ranges for VOL classification in the field might differ from those herein defined, even at 12 km·h^−1^, due to the fact that running events (footstrike and toe-off) are calculated differently when using an inertial measurement unit compared to 3D motion capture [20], and differences in running kinematics between treadmill and over ground exist [21].

Individualized training based on continuous real-time measurement of spontaneous running in a field environment could be undertaken with our proposed classification and use of inertial measurement units. Indeed, spontaneous changes in running form during racing or training could assist in identifying fatigue or changes in environmental conditions, for example, and allow a better understanding of runners [22]. By using such a device to objectively measure the DF, minor changes of the running form could be detected more readily than through the Volodalen^®^ scale, which also requires the presence of a Volodalen rater or coach.

A limitation to the present study exists. We confirmed the agreement between VOL and DF groups at only one speed (12 km·h^−1^). However, DF has been shown to decrease with increasing running speed (10 to 18 km·h^−1^) for both DF_low_ and DF_high_ groups [10]. Given that the classification of runners according to their V^®^score has been shown to be independent of running speed (10 to 18 km·h^−1^) [8], our defined DF ranges would merely shift with speed, indicating that the agreement between DF and VOL groups is generalizable across running speeds.

## 5. Conclusions

We identified a 79.8% (69.9%, 87.6%) agreement in VOL and the DF group classification of runners. In other words, the use of an objective DF measure was able to correctly classify a runner as a TER or AER runner based on the subjective Volodalen^®^ scale in 79.8% of cases. Our results suggest that the DF and V^®^score allow a similar classification of spontaneous running forms, particularly at 12 km·h^−1^, with a relatively high sensitivity (81.6% (68.0%, 91.2%)) and specificity (77.5% (61.6%, 89.2%)). At this speed, a DF greater than 28.8% would indicate a TER runner and one lower than 27.6% would indicate an AER runner. 

## Figures and Tables

**Table 1:** Participant characteristics for terrestrial runners (TER) and aerial runners (AER) (i.e., V^®^score (VOL) group), and high (DF_high_) and low (DF_low_) duty factor runners (i.e., duty factor (DF) group). Significant differences within groups are in bold font.

Group	VOL			DF		
Characteristics	TER	AER	*P*	DF_high_	DF_low_	*P*
Sex	M = 30; F = 19	M = 35; F = 5	NA	M = 30; F = 19	M = 35; F = 5	NA
Age (y)	38 ± 8	38 ± 10	0.976	39 ± 7	36 ± 11	0.384
Height (m)	1.72 ± 0.09	1.76 ± 0.07	0.013	1.73 ± 0.10	1.75 ± 0.06	0.398
Mass (kg)	68.4 ± 12.7	68.8 ± 7.5	0.412	68.1 ± 12.4	69.1 ± 7.9	0.936
Running time (h·week^−1^)	4.1 ± 2.5	4.9 ± 2.4	0.093	3.9 ± 2.1	5.1 ± 2.8	0.036
Running distance (km·week^−1^)	37 ± 23	47 ± 24	0.036	36 ± 23	47 ± 25	0.009
V^®^score	11.4 ± 2.5	19.0 ± 2.5	NA	12.8 ± 4.0	17.2 ± 4.0	<0.001
DF (%)	30.3 ± 1.8	27.8 ± 2.3	<0.001	30.8 ± 1.5	27.2 ± 1.6	NA

Note: Data are means ± S.D. DF: Duty factor, M: Male, F: Female, and NA: Not applicable.

**Table 2:** Number of terrestrial (TER) and aerial (AER) runners attributed to high (DF_high_) and low (DF_low_) duty factor groups.

DF Group	TER	AER
**DF_high_**	40	9
**DF_low_**	9	31

**Table 3:** Participant characteristics for terrestrial runners (TER) and aerial runners (AER) attributed to high (DF_high_) and low (DF_low_) duty factor groups.

Group	TER			AER		
Characteristics	DF_high_	DF_low_	*P*	DF_low_	DF_high_	*P*
Sex	M = 23; F = 17	M = 7; F = 2	NA	M = 28; F = 3	M = 7; F = 2	NA
Age (y)	38 ± 8	37 ± 9	0.708	37 ± 9	41 ± 13	0.296
Height (m)	1.72 ± 0.06	1.72 ± 0.06	0.900	1.76 ± 0.09	1.79 ± 0.08	0.401
Mass (kg)	68.8 ± 10.6	66.7 ± 6.7	0.928	68.2 ± 8.5	70.8 ± 10.6	0.369
Running time (h·week^−1^)	3.9 ± 2.8	5.0 ± 1.7	0.651	5.2 ± 2.1	3.9 ± 2.6	0.197
Running distance (km·week^−1^)	35 ± 27	46 ± 19	0.642	50 ± 21	33 ± 22	0.061
V^®^score	11.3 ± 4.6	12.0 ± 4.3	0.415	19.1 ± 4.5	18.6 ± 4.7	0.577
DF (%)	31.0 ± 3.4	26.4 ± 2.7	NA	25.8 ± 2.2	29.7 ± 3.7	NA

Note: Data are means ± S.D. M: Male, F: Female, and NA: statistical test not applicable.
